# Food insecurity and disasters: predicting disparities in total and first-time food pantry visits during the COVID-19 pandemic

**DOI:** 10.1007/s12571-022-01336-2

**Published:** 2022-12-24

**Authors:** Alexandria J. Drake, Lora A. Phillips, Brajesh Karna, Shakthi Bharathi Murugesan, Lily K. Villa, Nathan A. Smith

**Affiliations:** 1 School of Human Evolution and Social Change, Arizona State University, Tempe, AZ 85281 USA; 2 Knowledge Exchange for Resilience, School of Geographic Sciences and Urban Planning, Arizona State University, Tempe, AZ 85281 USA; 3 MAS-GIS Technical Support Services, Environmental Systems Research Institute Inc, Redlands, CA USA; 4 Phoenix Rescue Mission, Phoenix, AZ 85007 USA

**Keywords:** Urban Food Insecurity, Disasters, COVID-19, Arizona, Maricopa County, Food Deserts

## Abstract

In the U.S. state of Arizona, nearly one-third of households experienced food insecurity during the COVID-19 pandemic, an increase from one-fourth of households before the pandemic. Previous research on food insecurity in the wake of natural and human-instigated disasters demonstrates that groups vulnerable to food insecurity before a disaster are more susceptible to food insecurity during and after that disaster; however, less is known about whether this relationship also holds true during health-related disasters, such as the COVID-19 pandemic. We explore how the COVID-19 pandemic influenced urban food insecurity patterns by analyzing the socio-demographic characteristics of food pantry clients in Maricopa County, Arizona. Using data from Phoenix Rescue Mission (PRM), a local non-profit food service provider, two binomial logistic regression models compare the socio-demographic composition of total and first-time food pantry users before and during the pandemic. In addition to an overall increase in food pantry usage during the pandemic, we find that, while certain socio-demographic groups historically vulnerable to food insecurity experienced the predicted uptick in insecurity during the pandemic, other socio-demographic disparities were attenuated. These somewhat disparate findings illustrate the complex relationship between disasters and food insecurity in an urban context, offering several avenues for future research.

**Supplementary Information:**

The online version contains supplementary material available at 10.1007/s12571-022-01336-2.

## Introduction

### Food insecurity and socio-demographic inequities

According to the 1996 World Food Summit, “food security exists when all people, at all times, have physical and economic access to sufficient, safe and nutritious food to meet their dietary needs and food preferences for an active and healthy life” (World Food Summit Plan of Action, 1996).

Thus, in addition to basic food access, food security encompasses individuals having access to food that is culturally significant, while also taking personal preferences into account. Food insecurity is a great public health concern because lacking proper nutrition is associated with poor cognitive development in children (Alaimo et al., 2001), mental health issues (Bronte-Tinkew et al., 2007; Siefert et al., 2001), and an increased risk of chronic conditions like obesity and hypertension (Laraia, 2013), among other negative outcomes. Thus, disparities in those who experience food insecurity both reflect and exacerbate socio-demographic inequities that threaten racial/ethnic, gender, and socioeconomic justice, among other outcomes.

A large body of literature identifies which socio-demographic groups historically lack food security or, in other words, are the most food insecure. Food insecurity can be heavily influenced by income, as evidenced by the rate of food insecurity among low-income households, which is more than double the United States average (Coleman-Jensen et al., 2016; United States Department of Agriculture Economic Research Service, 2019). Food insecurity can highlight long-standing structural inequalities in the U.S. as certain demographic groups may be more vulnerable to not having access to nutrient-rich foods. In the United States, Hispanic, Black, and Indigenous individuals frequently experience high rates of food insecurity compared to other racial and ethnic groups (Myers & Painter, 2017; Odoms-Young, 2018; Phojanakong et al., 2019; Walker et al., 2021). Women also have an increased likelihood of facing food insecurity, but poverty rates can moderate this relationship (Broussard, 2019; Ma et al., 2021).

Socio-demographic differences in rates of food insecurity exist for a multitude of reasons. Socioeconomic status is inversely related to food insecurity, with poor and lower-income households relatively more likely to experience food insecurity (Gundersen et al., 2011). This relationship operates partially as a function of the fact that poor and lower-income households experience higher rates of unemployment, underemployment, and precarious employment, contributing to economic instability and deprivation that limits economic access to food. Similarly, previous work on the association between race/ethnicity and food insecurity demonstrates that racial/ethnic minority groups are more likely to face food insecurity (Healthy People 2020, 2022), in part because of structural barriers limiting employment opportunities, asset ownership, and access to mainstream credit options (Nam et al., 2015). For instance, in 2021, 8.1% of White Non-Hispanic Americans experienced food insecurity, compared to 15.8% of Hispanic/Latinos; 19.3% of Black, Non-Hispanics; and 23.5% of Indigenous Americans (Feeding America, 2021). Research in North America has also shown that women in non-married households face greater food insecurity, relative to both women in married households and men in non-married households, due to the relationship between this socio-demographic status and socioeconomic status (Matheson & McIntyre, 2014). Thus, common themes surrounding socio-demographic correlates of food insecurity in the United States context revolve around structural inequities faced by racial/ethnic minorities, gender-diverse individuals, and those living in poverty, among other at-risk groups (Hernandez et al., 2017a, 2017b; Whittle et al., 2015).

### Food insecurity during disasters

While food insecurity has recently declined in the U.S. (United States Department of Agriculture Economic Research Service, 2019) prior research shows an association between various types of disasters and heightened food insecurity. For instance, food insecurity and food pantry use rose significantly in the wake of Hurricane Katrina (Colten et al., 2008; Pyles et al., 2008), as well as during the Great Recession of 2007–2009 (Coleman-Jensen, 2012; Shackman et al., 2015; Slack & Myers, 2014). Such heightened food insecurity often persists past the officially designated end of a disaster, as disaster-affected economic structures work to recover (Shackman et al., 2015). Even several years after Hurricane Katrina, rates of food insecurity in Louisiana and Mississippi remained higher than before Katrina (Clay et al., 2018).

Prior research also shows that increases in food insecurity during disasters are driven, primarily, by growing food insecurity among the socio-demographic groups already facing high rates of food insecurity–whether due to households within these groups becoming newly food insecure or due to already food insecure households within these groups experiencing an even deeper need (Clay et al., 2018). In part, this reflects that socio-demographic groups at increased risk for food insecurity are, often, also the groups that face heightened risks during disasters. This heightened risk can stem from factors such as inadequate disaster preparedness, embodying conditions that amplify vulnerability, and/or structural inadequacies that cause these groups to fall through the cracks of emergency response (Ablah et al., 2009; Bethel et al., 2011; Golem et al., 2016). Looking at vulnerable populations after a hurricane, Clay and Ross (2020) find that economic instability, housing insecurity, and identifying as Black or Hispanic are risk factors for increased food insecurity (Gundersen & Ziliak, 2018). During the Great Recession, many food pantry users were seniors, were unemployed, did not have college degrees, and/or were not married (i.e., divorced, separated, or widowed) (Heflin & Price, 2019).

### Food insecurity and COVID-19

Thus, when considering disasters like Hurricane Katrina and the Great Recession, there is a clear pattern of growing food insecurity among the most historically food insecure socio-demographic groups. However, is the relationship between food insecurity and disasters consistent across all disasters and disaster types? The COVID-19 pandemic provides a platform to test whether a different type of disaster–a health disaster– likewise increases food insecurity among the most historically vulnerable urban populations.

As of July 2022, the COVID-19 pandemic has taken the lives of over 1 million people in the United States (Centers for Disease Control and Prevention, 2022) and 6.4 million globally (World Health Organization, 2022). Research prospectively anticipating food insecurity during the COVID-19 pandemic predicted 17 million newly food-insecure Americans due to the pandemic, for a total of 54 million food-insecure individuals (Gundersen et al., 2021). Additionally, nascent research demonstrates that certain historically vulnerable groups–such as low-income households and racial/ethnic minorities–experienced heightened food insecurity during the pandemic (Feeding America, 2021; Leddy et al., 2020; Pereira & Oliveira, 2020). However, little known research assesses the effects of the COVID-19 pandemic on urban food insecurity across a robust set of socio-demographic characteristics known to, historically, be associated with high levels of food insecurity. Beyond contributing to the extant literature, such knowledge would allow human services organizations to better prepare for (a) health-related disasters specifically and (b) disasters of all types more broadly, wherein many individuals lose physical and/or economic access to food (Mendez-Smith & Klee, 2020). During the first few months of the pandemic, Arizona in the United States had some of the highest infection rates in the world (Ailport, 2022). Given the extreme impact of the virus on Arizona residents, this research examines food insecurity in the context of data derived from a food pantry in Maricopa County, Arizona, to understand how this disaster impacted the socio-demographic distribution of food insecurity.

### COVID-19 and food insecurity in Maricopa County, Arizona, U.S.

Maricopa County had one of the earliest known cases of COVID-19 on U.S. soil, with a positive sample collected on January 22, 2022 (Scott et al., 2020). In response to the subsequent exponential growth in cases, on March 12, 2020, Arizona Governor Doug Ducey declared a public health emergency (COVID-19: Declaration of emergency, executive order, 2020). While deemed necessary for public health, these safety measures negatively impacted demand for agricultural products, petroleum products, manufactured goods, and labor across a variety of industries (Nicola et al., 2020). Additionally, many citizens responded by stockpiling food and healthcare products, which created strains on these industries (Nicola et al., 2020). Both the pandemic-related strain on food systems and the employment instability hastened by the state of emergency and stay-at-home order resulted in increased food insecurity in Maricopa County–an urban county of approximately 4.4 million people, home to the state capital of Phoenix, and the fourth most populous county in the United States (Greguska, 2020; United States Census Bureau, 2017, 2019). Given that Maricopa County had one of the earliest COVID-19 cases in the United States, the fact that Arizona embodied a COVID-19 hot spot-on multiple occasions during the pandemic (Ledesma et al., 2022; Longyear et al., 2020), and the socio-demographic diversity of Maricopa County, this location is a suitable site for exploring the relationship between COVID-19 and socio-demographic differences in food insecurity.

The pandemic-related uptick in food insecurity within Maricopa County can be contextualized using pre-pandemic food insecurity patterns within the state and county. Nationally speaking, between 2018 and 2020, an average of 10.7% of Americans were food insecure. In comparison, an average of 11.0% of Arizonians were food insecure over the same time period, making Arizona roughly reflective of national patterns. Relative to the nation and the state, however, food insecurity rates in Maricopa County trend a bit higher; a 2019 report identifies 13.7% of Maricopa County residents as food insecure (Brennan et al., 2019). Further, some Maricopa County residents are food insecure yet are not eligible for federal assistance programs, underscoring the crucial role that non-profit food service providers play in the county. For instance, many food insecure college students do not qualify for the Supplemental Nutrition Assistance Program (SNAP) because the Food Stamp Act of 1980 limits SNAP benefits for people enrolled more than half-time in a university, college, or technical school (Freudenberg et al., 2019; Larin, 2018). As such, an increased understanding of food insecurity in Maricopa County, including socio-demographic correlates before and during the pandemic, will allow for more targeted efforts to decrease food insecurity.

## Data and methods

To determine how the COVID-19 pandemic affected food insecurity across various urban socio-demographic groups in Maricopa County, Arizona, we provide a case study using de-identified, quantitative data provided by Phoenix Rescue Mission (PRM)–a non-profit that operates one brick-and-mortar and two mobile food pantries within Maricopa County, Arizona. Clients of PRM complete an extensive intake questionnaire administered by a trained PRM employee or volunteer, which is re-administered upon future visits to maintain data currency, thus providing date-stamped information on initial/continued food pantry use. This intake questionnaire includes detailed socio-demographic information on clients. The data constitute 28,724 pantry visits by 9,833 unique clients spanning the 6.5 months prior to the beginning of the COVID-19 pandemic (October 1, 2019-March 11, 2020) and the first 3.5 months of the COVID-19 pandemic (March 12, 2020-June 30, 2020), as demarcated by when Arizona Governor Doug Ducey issued the pandemic-related stay-at-home order ((COVID-19: Declaration of Emergency, Executive Order, 2020; Stay Home, Stay Healthy, Stay Connected, 2020). We supplement these data with aggregate client data over the same months in 2018 and 2019. The comparisons of 2018 and 2019 data allowed for verifying that variations in food pantry visits immediately prior to and during the pandemic are not reflective of regular seasonal changes (i.e.., PRM did not have an increase in visits during the spring and early summer every year). PRM changed its client intake process in October 2019, which is why the 6.5-month period was selected prior to the COVID-19 pandemic. The 3.5-month period after the pandemic began represented a time of general uncertainty, including the distribution of the first COVID-19 stimulus checks in April 2020 (USA.Gov, 2022). This also overlapped with state-mandated stay-at-home orders in Arizona, which lasted from March 31, 2020 to May 15, 2020, representing a period of peak vulnerability to food insecurity (Stay Home, Stay Healthy, Stay Connected, 2020; Stay Healthy, Return Smarter, Return Stronger, 2020).

Methodologically, we first provide a descriptive analysis of the number of total pantry visits month-by-month, divided between the pre-pandemic and pandemic periods. We then use t-tests to statistically compare the average monthly number of total pantry visits before and during the pandemic, as well as during the pandemic relative to prior years.

Next, we conduct two binomial logistic regressions to determine whether increases in the total number of pantry visits during the pandemic (Model 1) and increases in first-time pantry visits during the pandemic (Model 2) are primarily driven by increased demand among historically food insecure demographic groups. The dependent variable for both models is a categorical indicator of pre-pandemic visit (reference group) versus pandemic visit, with the key differentiating feature that Model 1 focuses on total pantry visits, while Model 2 focuses on first-time pantry visits.

The independent variables include age, household size, monthly gross income, gender, marital status, and race/ethnicity. For categorical variables (gender, marital status, and race/ethnicity), the reference group represents the historically less food insecure demographic group. As such, positive coefficients for independent variables indicate that groups historically facing greater food insecurity remained more likely to use food pantries during the pandemic relative to less food insecure groups, while negative coefficients indicate that the differences between more and less food insecure groups have attenuated during the pandemic. Specifically, gender is a categorical indicator of male (reference group), female, transgender, or undisclosed. Marital status is a categorical indicator of married or common-law marriage (reference group); single; separated, divorced, widowed; or undisclosed. Regarding race/ethnicity, PRM collects robust data that includes 37 races/ethnicities or combinations thereof. We aggregated these identifiers into 14 categories to ensure adequate group size and ease of interpretability: white alone (reference group); Black alone; Asian alone; Indigenous alone; Hispanic alone; Middle Eastern/North African alone; white alone, Hispanic; Black alone, Hispanic; mixed-race Black, non-Hispanic; mixed-race Black, Hispanic; mixed-race (but not Black), non-Hispanic; mixed-race (but not Black), Hispanic; other; and undisclosed. Descriptive statistics for the population of food pantry clients, pooled across the pre-pandemic and pandemic periods, can be found in Table 1.

**Table 1:** Descriptive Statistics for PRM Clients, October 2019 through June 2020

Variable	Total Visits	First-Time Visits
Mean Age	50.37	46.81
Mean Household Size	2.83	2.38
Mean Monthly Gross Income	$724.30	$602.42

Gender		
% Female	67.91%	69.39%
% Transgender	0.04%	0.03%
% Gender Undisclosed	0.24%	0.17%

Marital Status		
% Single	40.15%	45.42%
% Divorced, Separated, & Widowed	15.65%	10.96%
% Marital Status Undisclosed	4.25%	9.52%

Race/Ethnicity		
% Black alone	10.03%	10.97%
% Asian alone	2.23%	1.68%
% Indigenous alone	1.43%	1.71%
% Hispanic alone	53.88%	54.27%
% Middle Eastern/North African alone	4.96%	3.12%
% White alone, Hispanic	0.70%	0.64%
% Black alone, Hispanic	0.20%	0.18%
% Mixed-race Black, non-Hispanic	0.28%	0.25%
% Mixed-race Black, Hispanic	0.11%	0.07%
% Mixed-race, non-Black, non-Hispanic	0.45%	0.27%
% Mixed-race, non-Black, Hispanic	0.43%	0.38%
% Other	0.84%	0.72%
% Undisclosed	1.61%	2.17%

Source: Phoenix Rescue Mission

## Results

**Fig. 1:**
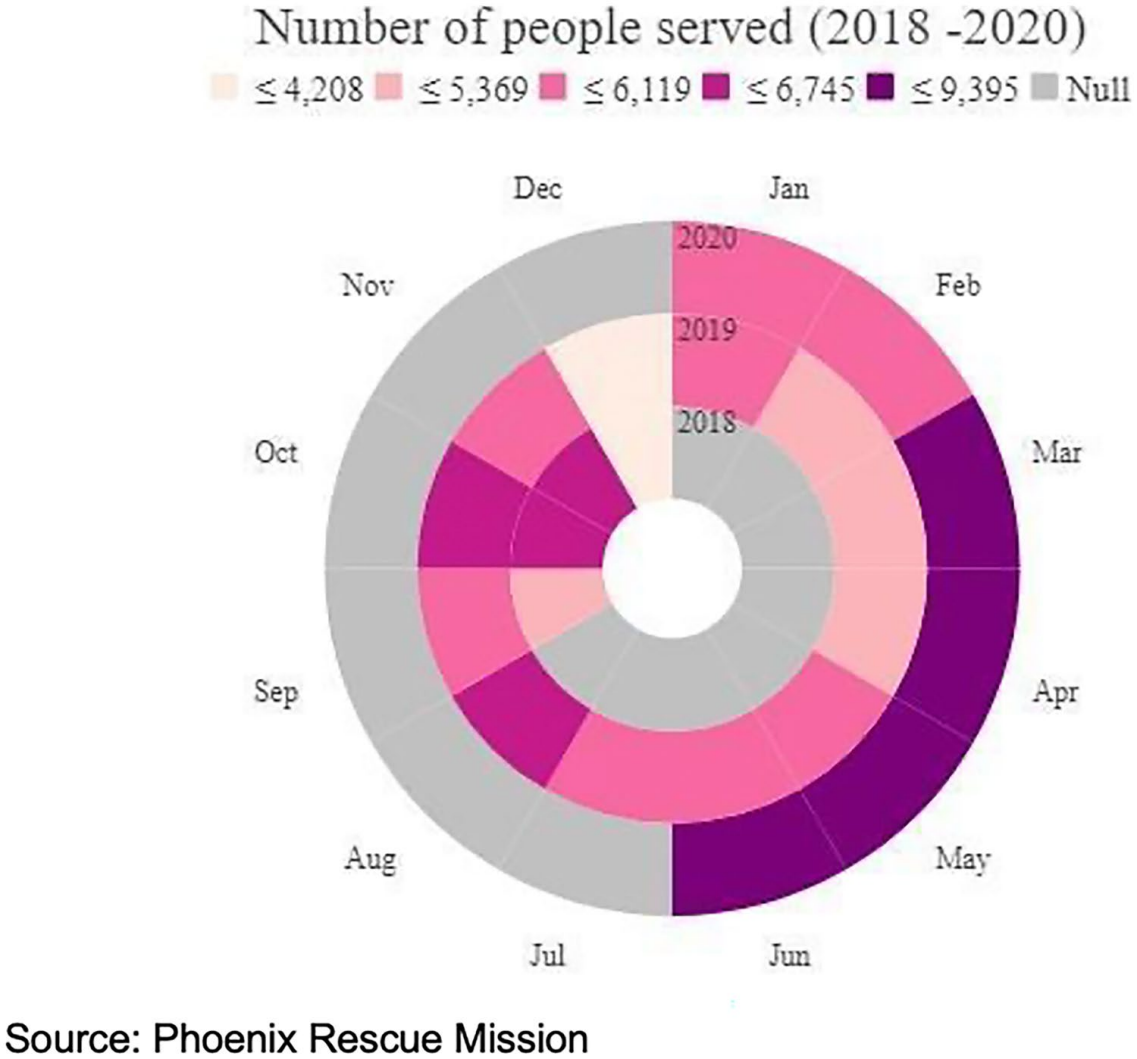
Number of clients served at a Phoenix Rescue Mission food pantry by month, 2018–2020. Source: Phoenix Rescue Mission

As shown in Fig. 1 and confirmed by t-tests, the total number of PRM food pantry visits during the pandemic period was significantly higher than both the period immediately preceding the pandemic (*p* < .001) and over the same period across prior years (*p* < .001). To unpack which groups drove this growth in food pantry use, we conducted two regressions, the results of which are shown in Table 2. Regarding total PRM pantry visits (Model 1), the odds of a client receiving food assistance during the pandemic decrease for each additional year of age (*p* < .001), for each additional household member (*p* < .001), and for each one dollar increase in monthly household income (*p* < .001), holding all other variables constant.

**Table 2:** Binomial Logistic Regression Predicting Total and First-Time Food Pantry Visits During the COVID-19 Pandemic

Time Period	Variable	Model 1 Coefficient (SE)	Model 2 Coefficient (SE)
Pre-Pandemic		(base outcome)	(base outcome)
Pandemic			
	Age	0.995***	0.981***
		(0.001)	(0.002)
	Household Size	0.941***	0.626***
		(0.006)	(0.012)
	Monthly Gross Income	0.999***	0.999***
		(0.000)	(0.000)

Gender			
	Female	1.031	1.088
		(0.028)	(0.059)
	Transgender	1.523	(omitted)
		(0.958)	
	Gender Undisclosed	0.490**	0.521
		(0.130)	(0.413)

Marital Status			
	Single	0.848***	0.649***
		(0.025)	(0.038)
	Divorced, Separated, & Widowed	0.841***	0.538***
		(0.032)	(0.051)
	Marital Status Undisclosed	1.305***	0.528***
		(0.086)	(0.052)

Race/Ethnicity			
	Black alone	0.973	1.053
		(0.046)	(0.104)
	Asian alone	1.466***	2.614***
		(0.124)	(0.474)
	Indigenous alone	0.984	0.797
		(0.105)	(0.188)
	Hispanic alone	1.616***	2.315***
		(0.051)	(0.153)
	Middle Eastern/North African alone	1.162*	0.877
		(0.071)	(0.144)
	White alone, Hispanic	1.375*	1.098
		(0.200)	(0.374)
	Black alone, Hispanic	0.734	0.829
		(0.209)	(0.542)
	Mixed-race Black, non-Hispanic	0.667	(omitted)
		(0.166)	
	Mixed-race Black, Hispanic	0.910	0.773
		(0.351)	(0.867)
	Mixed-race, non-Black, non-Hispanic	1.547*	0.878
		(0.279)	(0.489)
	Mixed-race, non-Black, Hispanic	1.379	1.136
		(0.263)	(0.503)
	Other	1.006	0.270*
		(0.138)	(0.142)
	Undisclosed	2.555***	3.917***
		(0.276)	(0.639)

Source: Phoenix Rescue Mission

* P < .05, ** P < .01, *** P < .001

Relative to men, the odds of women and transgender individuals receiving PRM food assistance during the pandemic were not significantly different; however, those who opted not to disclose their gender (N = 69) were significantly less likely than men to receive food assistance during the pandemic, holding all other variables constant (*p* < .01). The odds of PRM food pantry usage during the pandemic were also significantly lower among separated, divorced, or widowed clients (*p* < .001), as well as among single clients (*p* < .001) relative to married or in common-law marriage clients, holding all other variables constant. Converse to the results for gender, however, those who opted not to disclose their marital status (N = 1,221) had significantly higher odds of obtaining PRM food pantry services during the pandemic (*p* < .001), holding all other variables constant. Finally, relative to those clients identifying as white alone, the odds of PRM food pantry usage during the pandemic were significantly higher among those who identified as Asian alone (*p* < .001); Hispanic alone (*p* < .001); Middle Eastern/North African alone (*p* < .05); white alone, Hispanic (*p* < .05); mixed-race (but not Black), non-Hispanic (*p* < .05); or undisclosed (*p* < .001; N = 463), holding all other variables constant.

While Model 1 elucidates which socio-demographic groups most frequently received PRM food assistance during the pandemic, Model 2 assesses which socio-demographic groups demonstrated relatively high rates of newly seeking PRM food assistance during the pandemic by analyzing first-time visits.

As shown in Table 1, the odds of a client visiting a PRM pantry for the first time during the pandemic decrease for each additional year of age (*p* < .001), for each additional household member (*p* < .001), and for each one dollar increase in monthly household income (*p* < .001), holding all other variables constant. In terms of gender, the odds of receiving first-time food services from PRM during the pandemic are similar across gender groups relative to men, with the notable exception that all food pantry visits by transgender individuals occurred during the pre-pandemic period and, thus, were omitted from the regression model. There were also significant differences in the odds of first-time PRM pantry visits during the pandemic by marital status. Relative to those who were married or in a common-law marriage, the odds of a first-time PRM pantry visit were lower among those who were single (*p* < .001); divorced, separated, or widowed (*p* < .001); or did not disclose their marital status (*p* < .001), holding all other variables constant. Finally, relative to those clients who identified as white alone, the odds of going to a PRM food pantry for the first time during the pandemic were significantly higher among those who identified as Asian alone (*p* < .001), Hispanic alone (*p* < .001), or race/ethnicity undisclosed (*p* < .001), holding all other variables constant. On the other hand, the odds were significantly lower among those who identified as other race/ethnicity (*p* < .05), holding all other variables constant. Similar to the gender results, pantry clients who identified as mixed-race Black, and non-Hispanic visited for the first time entirely before the start of the pandemic and were omitted from the regression model.

## Discussion

This research assessed whether the COVID-19 pandemic increased food insecurity among the historically most food insecure socio-demographic groups. Using client intake data from PRM, our findings suggest varying degrees of conformity with the idea that disasters increase food insecurity among already food insecure groups. Aligning with prior research, several racial/ethnic minority groups were more likely to be food insecure both before and during the pandemic (Broussard, 2019; Dammann & Smith, 2010; Feeding America, 2021), and results were also in the expected direction for monthly income. Surprisingly, however, gender differences in food insecurity appear to have been attenuated during the pandemic, and individuals who were married or in a common-law partnership were more food insecure than those who were single or previously married. Further, older adults, members of larger households, and members of particular racial/ethnic minority groups were less food insecure than expected based on pre-pandemic relative rates. (Odoms-Young et al., 2009; Ziliak & Gundersen, 2020). Taken as a whole, these findings suggest disasters can increase food insecurity among some historically vulnerable urban groups who visit food pantries. However, differences in food insecurity may also be reduced between some historically more and less food insecure groups–perhaps due, in part, to historically less food insecure groups, such as white men, being drawn into the fold as they are exposed to the stressors associated with the disaster (Coleman-Jensen, 2012; Hanson et al., 2007).

Moreover, Arizona, as a whole, had a moderately lower level of food insecurity in 2021 (5.9%) than the most food insecure state in the United States (Arkansas- 7.1%), indicating that some vulnerable populations may have been insulated from increased food insecurity during the pandemic (Feeding America, 2021). This points to one limitation of our study; namely, our data are derived solely from one non-profit operating three food pantries within Maricopa County. Future research should ascertain whether the patterns we found are consistent across locales and across health disasters more broadly. In doing so, we encourage scholars to remain cognizant of the reality that particular socio-demographic groups experience barriers to accessing food pantries and, also, may prefer particular food pantries over others if, for instance, particular pantries provide individualized plans (Cooksey-Stowers et al., 2019; Martin et al., 2016). Related to this point, we secondly acknowledge that–although PRM has a demonstrated track record of reaching diverse socio-demographic groups through their use of both brick-and-mortar and mobile food pantries (author redacted) –we are employing food pantry usage as a proxy for the level of food insecurity across socio-demographic groups. Future research could explore the extent to which groups face food insecurity but either do not receive food assistance or receive food assistance through other channels. This point is particularly relevant in the wake of disasters such as the COVID-19 pandemic, when organizations and institutions that do not normally provide food assistance are more likely to supplement the efforts of food pantries through, for instance, initiatives like drive-through meal services (Arizona Department of Economic Security, 2021). Initiatives such as these may divert clientele from traditional food pantries; however, it is unclear whether particular socio-demographic groups are more likely than others to utilize emergent food channels as opposed to traditional food pantries. Third, our data are limited to only the COVID-19 health disaster and, due to the aims of our study, the first several months of the pandemic, when stay-at-home orders were in place. Future work might also explore food pantry usage for the entirety of the COVID-19 pandemic as well as the aftermath of the pandemic. More broadly, and in light of our findings herein, future research can make inroads into understanding both the impact of disasters on historically food secure socio-demographic groups, as well as the similarities and differences in vulnerability across different types of disasters, including why observed differences emerge.These limitations point to a pressing need for more widespread data collection and data sharing across the many food providers that serve local areas.

Despite these limitations, our findings offer actionable insights for food service providers. Broadly speaking, we demonstrate that food service providers should not assume that food pantry usage during the next disaster will be the same as during prior disasters, particularly regarding the socio-demographic groups who are affected. As such, we recommend that food service providers strive for organizational resilience–or the ability “to prepare for disruptions, to recover from shocks and stresses, and to adapt and grow from a disruptive experience”–to ensure that food distribution will stably continue in the face of a diverse range of disasters that may affect socio-demographic groups differently (Rodin, 2014, p. 3). Organizational resilience building is particularly imperative given evidence that, during the pandemic, food pantries in Europe and the United Kingdom experienced problems with transporting food to increased food insecure populations (Dekkinga et al., 2022); ineffective communication and integration between other charities and food distribution systems; lower nutritional quality food; financial hardship; and difficulty distributing food due to social distancing (Barker & Russell, 2020).

Despite the reality that PRM faced the same challenges to reaching food insecure populations in Maricopa County, according to Nathan Smith, the Chief Program Officer of PRM, “no days of food distribution service were missed because of the pandemic at a time when the demand for services had increased beyond anything that [we] had ever experienced (N. Smith, personal communication, February 26, 2021).” In light of PRM’s relative success in addressing these challenges, particularly in the context of our findings which highlight a need to build organizational resilience to ensure adequate responses to the diverse effects of disasters on food insecurity, we offer policy and programmatic insights that may aid other food service providers using lessons learned from PRM. Indeed, PRM was so successful in addressing these challenges, in part, because it embodied multiple characteristics of resilient communities and organizations. Following several components of resilience highlighted by Judith Rodin (Rodin, 2014), PRM demonstrated awareness and adaptability by adjusting protocol to limit catastrophic shocks (i.e., curbside pantry pickup, enhanced sanitation measures, conducting the intake form on cell phones outside the building rather than on computers inside), ensuring that vulnerabilities did not become liabilities. PRM also showed characteristics of diversity and integration by turning to different sources of capacity to meet client needs. This included reaching out to new donors (diversity) to ensure that they had enough food to meet the needs of individuals and families visiting the food pantry. And since PRM has established itself as a reliable provider of quality food in Maricopa County, they were able to draw on a broader network to receive grants from the Governor's office, private foundations, and other corporations (integrated). The combination of these multiple efforts showed PRM to be a self-regulating organization that could take on a catastrophic shock to the system and still meet community needs. The capacity for PRM to serve as a resilient community organization during the COVID-19 pandemic was vital in meeting the needs of those who were already food insecure, even as the pandemic further exacerbated their food insecurity, as well as meeting the needs of groups that may have been newly food insecure from the pandemic.

To understand how food pantries worldwide could more effectively address food insecurity during a disaster, Smith stated that urban food pantries should prepare by preemptively developing a disaster preparedness plan. Given the findings herein, such a plan should include an extensive marketing plan for diverse socio-demographic groups, aimed at increasing access for the most food insecure groups. Such a plan should also include the ability to repurpose and increase staff due to a loss in volunteers and staffing during a disaster, an emergency fund to bolster increasing costs during a disaster, diverse food sources, and a plan to bolster staff morale during the crisis. Based on our research, we add that urban food pantries should not only be prepared to serve their traditional clientele but also to manage declining or increasing demand across a range of socio-demographic groups, given the increased food insecurity among less food insecure populations, such as white men. It is also essential to take into consideration that food security does not simply mean having food available. When focusing on food security in times of normalcy and disaster, food pantries should, as much as possible, focus on making sure the items given to clients meet their cultural, age, health, personal preference, and other food needs.

Policy change can also reduce food insecurity during disasters, including global pandemics. For example, creating economic stimulus policies, expanding the income and eligibility criteria for public food assistance, increasing food distribution in the highest need areas, and expanding international trade policies have been shown to be effective (Barlow, 2020; Laska et al., 2020). Arguably, however, the most effective policy changes will be proactive rather than reactive; namely, while temporary policy changes in the wake of disasters are essential for ameliorating the disaster’s worst harms, permanent policy changes must reduce the structural inequities that make particular socio-demographic groups both more food insecure and more vulnerable in the wake of disasters. This research, thus, offers valuable, actionable insights into addressing food insecurity during health disasters such as the COVID-19 pandemic. While such disasters may not always be preventable, by understanding the socio-demographic groups most vulnerable during disasters, coupled with insights from resilient food service providers such as Smith, food insecurity during disasters can be proactively reduced (Bazerghi et al., 2016; Penco et al., 2021).

## Conclusion

Food insecurity poses a significant threat to mental and physical health and well-being globally, and food insecurity is exacerbated by disasters (Fang et al., 2021; Wolfson & Leung, 2020). Prior research demonstrates that, during natural and human-instigated disasters, the most food insecure socio-demographic groups experience increased food insecurity. We are among the first to test whether a different type of disaster–a health disaster–demonstrates the same pattern. Assessing the effects of the COVID-19 pandemic on urban food insecurity across a robust set of socio-demographic characteristics, we found that the COVID-19 pandemic was associated with increased food insecurity among some of the most historically food insecure socio-demographic groups; however, inconsistent with the literature on natural and human-instigated disasters, we also found that disparities in food insecurity were attenuated between particular socio-demographic groups, implying growing food insecurity among historically insulated socio-demographic groups. These findings elucidate the reality that the relationship between food insecurity and disasters is not straightforward but, rather, varies depending upon existing vulnerabilities to food insecurity, the specific vulnerabilities associated with a particular disaster, and the interplay between the two. Beyond contributing to scholarship on food insecurity broadly and food insecurity during disasters specifically, this research should inform the practices of food service providers and policies meant to ameliorate food insecurity.

## Supplementary Information

Below is the link to the electronic supplementary material.

Supplementary file1 (XLSX 1499 KB)

## Data Availability

The participants of this study did not give written consent for their data to be shared publicly, therefore the research support data is not available.
